# Fracture and mechanical properties of an impact toughened polypropylene composite: modification for automotive dashboard-airbag application

**DOI:** 10.1039/d3ra04151d

**Published:** 2023-09-13

**Authors:** Saiful Bahri Mohd Yasin, Joseph S. Terry, Ambrose C. Taylor

**Affiliations:** a Mechanical Engineering Department, Imperial College London, South Kensington Campus London SW7 2AZ UK s.mohd-yasin21@imperial.ac.uk saiful926@uitm.edu.my; b Faculty of Applied Sciences, Universiti Teknologi MARA Cawangan Perlis Kampus Arau 02600 Arau Perlis Malaysia

## Abstract

Thermoplastic olefin (TPO) is the principal material for automotive instrument panels and is prone to fracture especially under heavy airbag deployment, which can prevent the airbag deploying properly. Thus, polyolefin elastomer (POE) was introduced to improve impact properties and fracture resistance. Fundamental methods to characterise TPO with the addition of POE are proposed. The influence of POE content on the mechanical properties was examined. With increasing POE content, the storage modulus and glass transition temperature values decreased, and the damping increased due to the POE increasing the polymer chain mobility. The tensile modulus, ultimate tensile strength and yield stress decreased with increasing POE content, while the ductility of the blends significantly increased. Furthermore, the POE reduced hardness and increased energy absorption during impact. In the fracture analysis, the addition of POE content increased the fracture resistance by increasing the crack energy and decreasing the resistance to crack initiation. Fractographic analysis showed how stretched microfibrils in the blends increase the fracture resistance. These results gave a significant indication of the utility of the elastomer in improving some mechanical properties and impact toughness of the interior automotive material to resist an undesired failure or over-fracture in airbag deployment.

## Abstract 2

Thermoplastic olefin (TPO) is the principal material for automotive instrument panels and is prone to fracture especially under heavy airbag deployment, which can prevent the airbag deploying properly.

## Graphical abstract

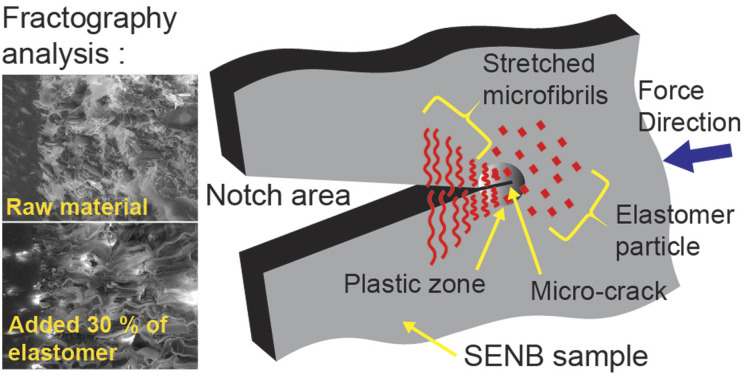

## 1. Introduction

The use of elastomers, including polypropylene, for impact modification in material engineering applications arose from material developments in the mid-1990s and early 2000s. One of the main uses of impact modified materials is the automotive industry.^1^ Thermoplastic olefin (TPO) and thermoplastic elastomer (TPE) are polypropylene–elastomer blends and are commonly used in the automotive industry especially since there is a requirement to absorb the impact of a human on internal vehicle structures during a car collision to reduce severe injuries and fatality. Airbags deploy when a significant impact is detected. For a car passenger, the airbag is housed in the instrument panel. This panel fractures along pre-cut lines when the airbag deploys. The automotive instrument panel (IP) and airbag housing are applications that use elastomer as one of the main ingredients, although there is a lack of research on how this IP-airbag structure relates to automotive safety.

**Fig. 1:**
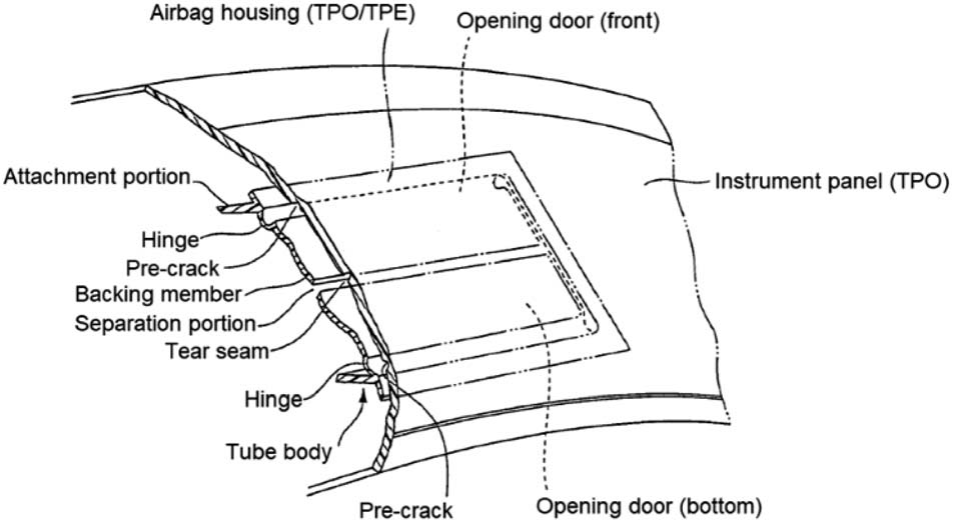
Cross-section of automotive instrument panel showing airbag structure.^4^ (The text and drawings of a US patent are typically not subject to copyright restrictions).

In their review of airbags,^2^ Nayak *et al.* emphasized that airbag technology is undergoing a continual revolution in terms of design, materials and performance, but is failing to address the use of elastomer in selecting materials for the airbag housing. While, in a review on the thermoplastic polyolefin elastomer to bridge the performance gap between rubber and thermoplastic,^3^ the use of elastomer in TPO will provide a synergistic effect and behave like a crosslinked rubber with the processing flexibility of a thermoplastic, thus it is suitable to use TPO as an internal automotive material to absorb human body impacts. The patent by Nishijima *et al.*^4^ confirms that an instrument panel covers the airbag housing, and quoted their material selection where the airbag housing is made of either TPO or TPE (the selection of material depends on the type of airbag), and the instrument panel (IP) is made of TPO (a polypropylene composite). The TPO used for IP is prone to fracture under an impact during airbag deployment as this material is designed to meet requirements such as good impact resistance, dimensional stability, weathering resistance and not fracture properties. Thus, POE is introduced to improve material flexibility and give better fracture resistance, especially against a heavy impact during airbag deployment. Based on the previous finding on the fracture behaviour, Sugaya *et al.*^5^ reported the development of plastic fracture technology for passenger airbag tear seams to increase the accuracy of the tear fracture process at low temperatures during airbag deployment. However, the most critical region during airbag deployment is the pre-crack areas (see Fig. 1) which determine whether the airbag deploys at the correct angle, and the literature does not report this crack behaviour.

Material modification such as the use of polypropylene (PP) and elastomer has been driven by research showing that they offer exceptional mechanical and physical properties toward failure resistance, such as excellent low-temperature, impact strength, improved rheological and moulding processability.^6,7^ The use of POE in PP or TPO was introduced in 1993,^8^ and since then it is extensively used in many applications due to the flexibility of the material and ease of mixing with other fillers.^9–13^

Zhao *et al.*^14^ reported that the use of POE in PP gave improved impact toughness and flexibility, particularly increasing the strain to failure by more than 1000% with a POE content above 30 wt%. The tensile strength decreased by 7% to 45% as the POE content was increased from 10 wt% to 50 wt%. Jiao *et al.*^15^ indicate that 10 wt% to 40 wt% POE in the PP matrix provides a dual function by reinforcing and toughening. Chum *et al.*^6^ and Silvis *et al.*^7^ state that TPO containing 30 wt% of POE offered excellent impact properties at low temperatures. The long chain branching structure of POE improves its dispersion in the TPO matrix thus enhancing the impact toughening properties. However, Carriere and Silvis^16^ and Muñoz-Pascual *et al.*^17^ reported that the increment of POE in polypropylene matrix will rapidly decrease the interfacial tension, thus increasing the compatibility^16^ and reducing the stiffness of the material.^17^ On the other hand, Da Silva *et al.*^18^ report that the use of POE will provide better processability compared to other impact modifiers, thus offering a significant cost advantage. The fracture behaviour was reported by Yin *et al.*^19^ and Fasce *et al.*,^20^ who showed the behaviour varied with the composition of the blends, from unstable crack growth in the case of the PP homopolymer under impact loading to increasingly more stable crack growth in the case of the blends,^20^ thus elastomer modification in the polypropylene matrix shifted the fracture pattern due to strong shear yielding of the matrix between micro-voids which took place after the brittle–ductile transition,^19^ thus increasing the crack propagation energy and decreasing resistance to crack initiation. A similar report has been found about the PP matrix containing POE in tensile high-speed test,^21^ where the samples with POE contents of 35 wt% and above will deform at the notch region without fracture due to the shift of the brittle–ductile transition point. Huang *et al.*^22^ indicated that polyolefins and polymer blends tend to exhibit stable crack growth. These materials are best characterized by elastic plastic fracture mechanics using crack growth resistance (*J*–*R*) curves. Researchers tend to use scanning electron microscopy (SEM) to study the fracture surface in materials and to gain a deeper understanding of the fracture process.^19,23^

The aim of the present study is to characterise the fracture properties of TPO used for the IP material and TPO modified with POE. This was used to develop an appropriate experimental methodology for determining the fracture properties of the IP-airbag structure. Furthermore, fundamental experiments such as tensile and impact tests were conducted to understand the properties of the modified material, and to detect potential problems during processing or material application. Lastly, the relations generated by these experiments will be examined to gain additional insights into material behaviour, designing process and structural integrity in the IP-airbag application.

## 2. Theoretical fracture approach

For polymers which are linear elastic, and hence are relatively brittle, the methodology for determining fracture toughness (*K*_IC_) and fracture energy (*G*_IC_)^24,25^ is well established and universally accepted. Measuring these properties remains more challenging for tough polymers which exhibit nonlinear behaviour. The J-contour integral is one approach used to measure the fracture resistance (*J*) for tough nonlinear materials such as metals and polymers:^26^1
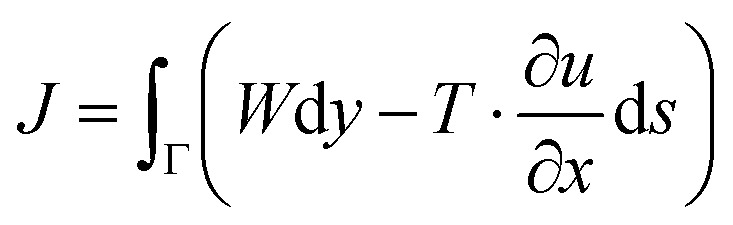
where *J* is the J-integral, *W* is the strain-energy density, *Γ* is a curve surrounding the notch tip, *T* is the traction vector at the outside of *Γ*, *x* and *y* are in-plane coordinates, *u* is the displacement vector and ds is an increment of the length along the contour *Γ*.

Experimentally, the J-integral can be determined as a function of crack extension, Δ*a*, in a single-edge notched bend (SENB) test for a crack loaded in tension.^27^ The European Structural Integrity Society (ESIS) TC4 J-protocol^28^ and the American Society for Testing and Materials (ASTM) standard D6068-10 ^29^ have proposed a robust methodology for determining the fracture resistance of tough polymers using multiple specimens. *J* is measured for several crack growth lengths by loading a series of identical specimens to various sub-critical displacements.^30^ A value of *J*, termed *J*_c_, for each specimen can be calculated using:2
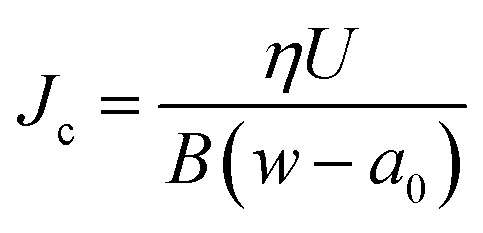
where *η* is the shape factor, taken as 2 for SENB specimens,^28^*U* is the energy absorbed during the test, calculated using the area under the load–displacement graph, *B* is the specimen thickness, *w* is the width, and *a*_0_ is the length of the pre-crack. The specimen dimensions are selected to promote plane strain conditions and so achieve conservative fracture resistance values. For *J*_c_ values to be valid, the crack growth (Δ*a*) must lie between Δ*a*_min_ = 0.05 mm, and Δ, which is calculated using:

3 Δ*a*_max_ = 0.1 (*w* − *a*_0_)

The term *J*_0.2_ is commonly used to define the fracture parameter in *J*_c_ analysis. This is determined by applying a power law fit to the *J versus* Δ*a* graph, then taking the value at Δ*a* = 0.2 mm of crack growth.

To increase the accuracy of the power law fit, it is recommended that *J*_c_ values are calculated over well-distributed Δ*a* values and at least seven repeats are performed, with two Δ*a* values falling between 0.2 mm and 0.4 mm,^28^ so:

4 *J*_c_ = *X*Δ*a*^*Y*^

5 *J*_0.2_ = *X* (0.2)^*Y*^

The distribution results for the power law fit and fracture resistance (*J*_0.2_) are highly influenced by the method used to determine the crack length. Samples must be fully fractured post-test to measure properly the crack growth, and samples may exhibit unstable crack length. Both factors can make it difficult to accurately calculate the average crack growth. Staining can be applied to the cracked surface post-testing to aid this measurement.^23^

## 3. Material and methods

### 3.1. Introduction

**Fig. 2:**
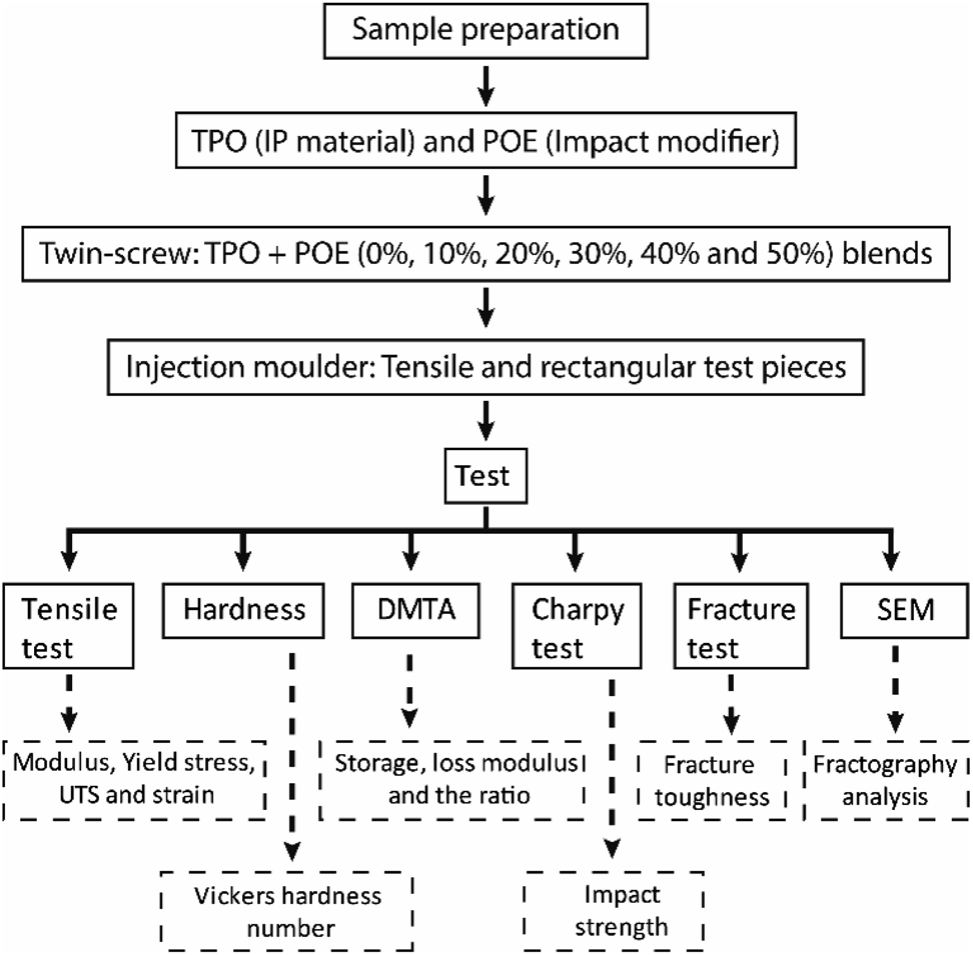
Workflow of sample preparation and testing.

Prior to evaluating the fracture behaviour, it is necessary to characterise the physical and mechanical properties of the materials. An overview of the material preparation and testing stages is shown in Fig. 2. The procedures are divided into sample preparation and mechanical tests.

### 3.2. Sample preparation

**Fig. 3:**
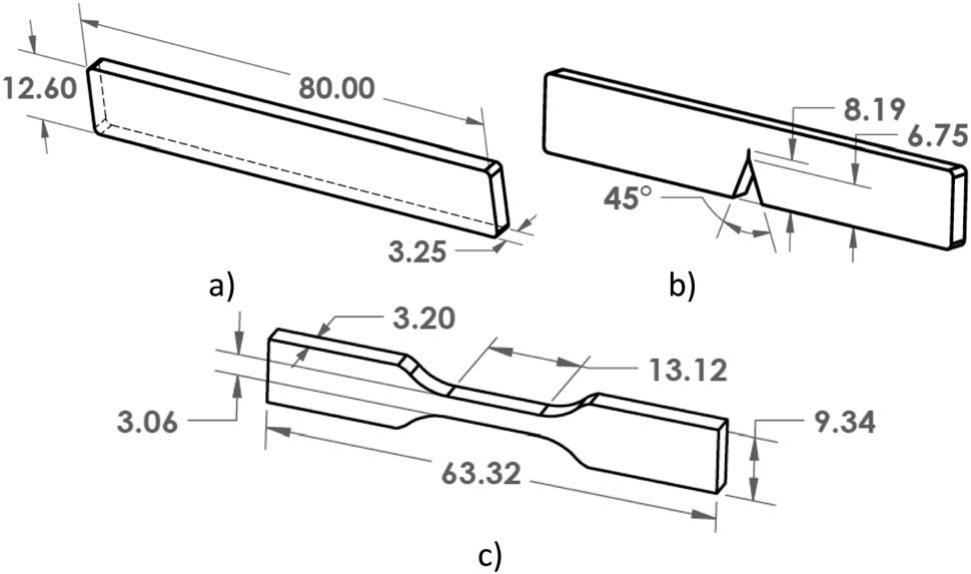
Rectangular (a), single-edge notch bend – rectangular modification (b) and tensile (c) test pieces.

A commercial grade of thermoplastic olefin (TPO) was investigated and modified with polyolefin elastomer (POE). The TPO is a high flow 25% talc filled ethylene propylene diene monomer (EPDM) rubber modified polypropylene compound. To understand the properties and morphologies of the material blend in enhance mechanical and fracture properties, the research commenced by testing both neat TPO and TPO blended with varying amounts of POE, ranging from 10% to 50% (Eurolab 16, Thermo Scientific). A mixture of TPO and POE granules was poured into the hopper and the barrel temperature zones were set at 180 to 200 °C. A screw speed of 20 rpm and a 4 mm diameter die were used. A pelletizer (Thermo Scientific) set at 2 rpm was used to cut the extrudate into 2 mm-long pieces. To produce tensile, single-edge notch bend (SENB) and rectangular test pieces (see Fig. 3), the dried granules were placed into a hydraulic injection moulder (Minijet Pro, Haake). The melt temperature was set to 200 °C, with a mould temperature of 40 °C. For tensile and rectangular test pieces, a mass of 3 g and 5 g respectively were required. The materials were heated for 3 minutes and 5 minutes respectively, then injected into the mould and a hold pressure applied for 1 minute. The mould was then opened and the specimen was allowed to cool at ambient conditions.

### 3.3. Material characterisation and measurement

#### 3.3.1. Tensile test

Quasi-static tensile tests on TPO, POE and TPO–POE blends were performed using a 50 kN universal testing machine (3369, Instron). A crosshead speed of 100 mm min^−1^ was used, and the strain was measured using an optical extensometer (2663-901, Instron). Two dots were marked on the test piece for extensometer measurement, and five replicates were used. The tensile modulus was calculated over an interval of 0.05% to 0.25% strain. The yield stress (use 0.5% offset yield stress), ultimate tensile strength and strain to failure were also calculated.

#### 3.3.2. Dynamic mechanical thermal analysis (DMTA)

The viscoelastic response of the TPO, POE and TPO–POE blends were characterised using a dynamic mechanical analyser (Q800, TA Instruments). Rectangular specimens of 60 mm × 12.6 mm × 3.25 mm were tested in dual cantilever mode with a 30 μm maximum displacement at a single frequency of 1 Hz. The temperature dependence of the material was determined from −95 °C to 180 °C at a ramp rate of 5 °C min^−1^. The storage modulus (*E*′), glass transition temperature (*T*_g_) and damping tan *δ* were measured for each material formulation.

#### 3.3.3. Charpy impact test

The impact toughness of TPO and TPO–POE blends was measured by the Charpy impact test using a pendulum impact tester (5102.100, Zwick). The rectangular samples of 80 mm × 12.6 mm × 3.25 mm with a V-notch were used to measure the energy absorption under a 2 J hammer impact load. The energy taken to rupture the sample was recorded.

#### 3.3.4. Hardness test

A hardness testing machine (114139, ZwickRoell) was used to the measure Vickers hardness number (VHN) of 5 rectangular samples for each formulation. The force setting H_v5_ was selected to apply 5 kg of load on the material surface for 10 seconds using a pyramidal diamond tip. The perpendicular diagonal lengths (*d*_1_ and *d*_2_) were measured using an optical microscope (Axio Scope A1, Zeiss) and averaged to calculate *d*. The hardness index is calculated using:6
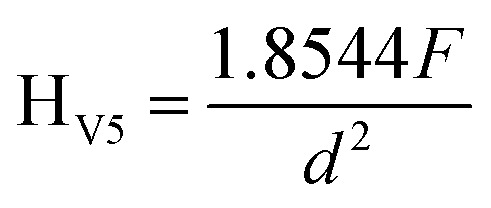
where *F* is load (kgf) and *d* is the mean diagonal length (mm).

#### 3.3.5. Fracture test

SENB samples with dimensions 80 mm × 12.6 mm × 3.25 mm were prepared to obtain a different amount of crack growth and the fracture energy of the TPO and TPO–POE blends under quasi-static conditions. The pre-notch was machined using a horizontal mill to produce 45° pre-notches of the required depth, see Fig. 3. The pre-crack was prepared by sliding a fresh razor blade across the notch tip for each specimen to generate a sharp and repeatable crack tip. A three-point bending fixture was used on a universal testing machine (3366, Instron) to perform fracture and compliance tests. Tests were performed under ambient temperature (22 °C) at a crosshead speed of 1 mm min^−1^.

After the testing, the notch region was stained using green luminescent ink (Art. Nr. 48864-1, Staedtler) to detect final crack growth, and drying was speeded up using a hairdryer. Polypropylene materials tend to craze,^28,31^ forming white marks on the fracture surface. The ink makes the crack growth and stress whitening on the fracture surface easier to identify. The samples were broken cryogenically to ensure no additional crack growth occurred. Images of the surface of the pre-crack and final crack were captured using an optical microscope (SMZ800, Nikon) and measured in image-J software. The fracture resistance was calculated using J-code programming in Matlab software. To develop the *J*_c_*versus* Δ*a*_max_ graph required at least 7 samples to be tested.

#### 3.3.6. Scanning electron microscopy (SEM)

Scanning electron microscopy (SEM) (Mira, Tescan) was utilized to investigate the post-failure fracture surface of the TPO and TPO–POE blends. The area of interest on the fracture surface for fractographic analysis is the onset of fracture to investigate the stretched microfibrils and to observe the micro-voids of each specimen. To examine the microfibrils on the fracture surface, the SEM was operated with an energy of 25 keV and a beam current of 300 pA. For the observation of microcracks and voids, both settings were adjusted to 1 keV and 30 pA each. The scanning process requires that the specimen surface be conductive, thus a sputter coater machine (Q150+, Quorum) was used to apply a 5 nm layer of chromium to coat the entire fracture surface.

## 4. Results and discussion

### 4.1. Introduction

Blends of TPO and POE with 0% to 50% POE were used. To obtain the fundamental characterisation for TPO, TPO–POE blends and POE, the testing used tensile, DMTA, Charpy, hardness, SEM and fracture tests.

### 4.2. Tensile test

**Fig. 4:**
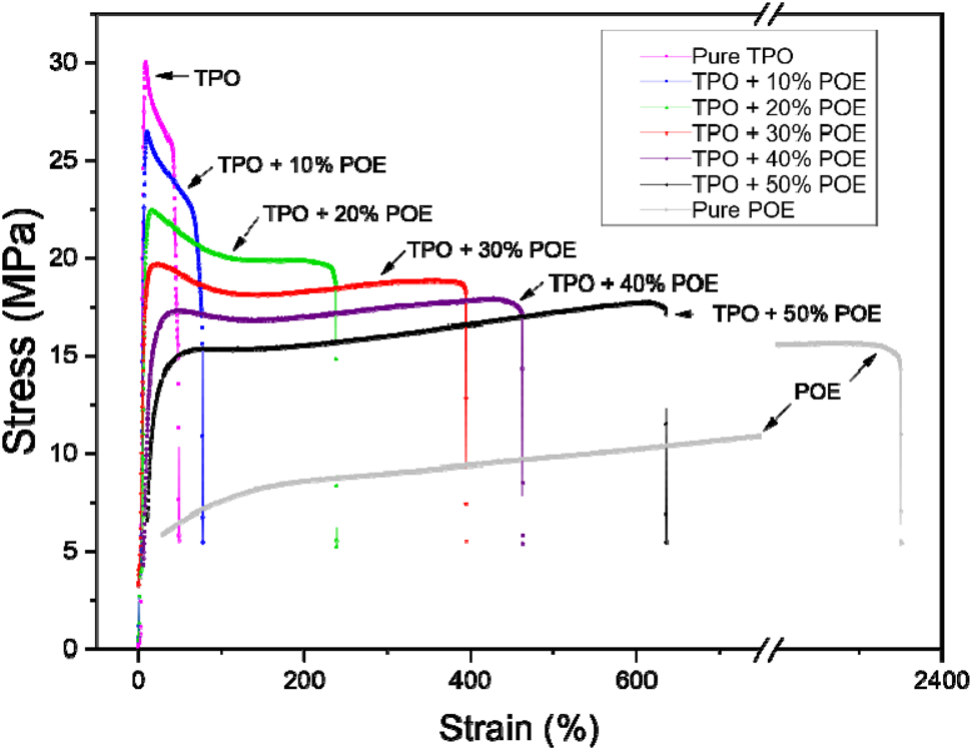
Tensile stress *versus* strain curves for TPO, POE and TPO–POE blends.

Fig. 4 shows representative stress–strain curves, for TPO, POE and TPO–POE blends. The main observation from the curves is that the plastic deformation increases with increasing POE content, while the yield stress and ultimate tensile strength decrease accordingly. The TPO–POE blends with a POE content below 20 wt% exhibit a high yield strength and almost reach the ultimate tensile strength with noticeable necking during the tensile test. However, at 30 wt% of POE content, the tensile strength is nearly equivalent to the yield strength, and the strength surpasses the yield region after the addition of POE at 40 wt% in TPO. While, at 50 wt% of POE content, the necking disappeared and the curve shows an increment of strain hardening after the yield point. The increment of strain hardening on the curve is detected after 150% strain until the fracture point. The increment of strain hardening for TPO–POE blends above 30% is found to have a similar trend with the stress strain curve for neat POE. Thus, this signifies that the POE compositions of 30 wt% and above provide a synergistic effect on tensile properties between both materials.

**Fig. 5:**
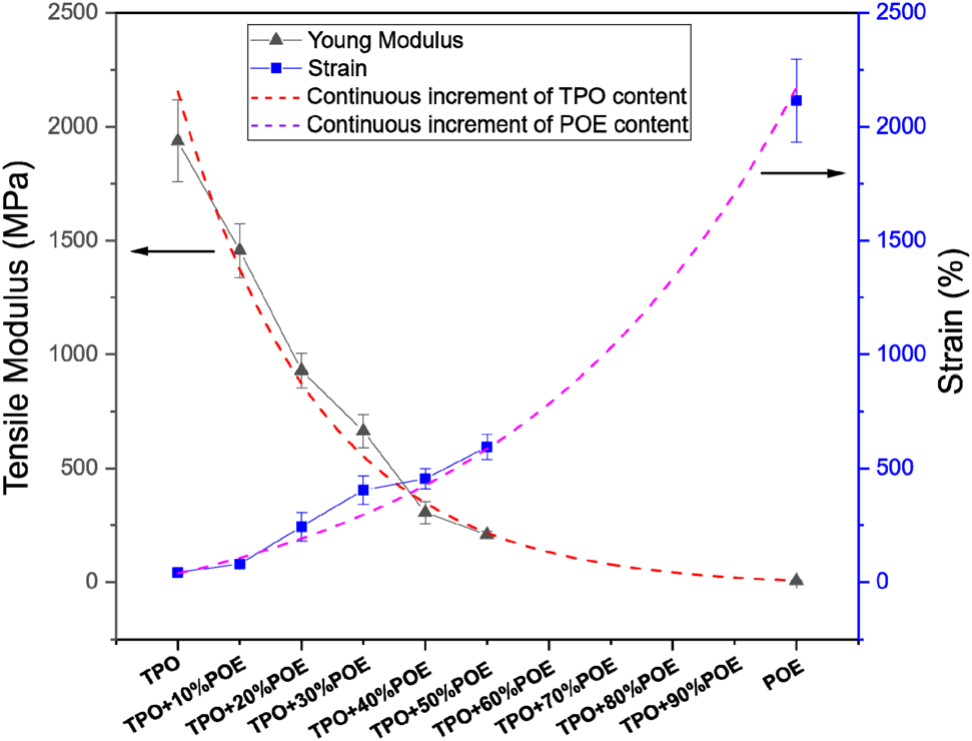
Tensile modulus and strain to failure for TPO, POE and TPO–POE blends.

**Fig. 6:**
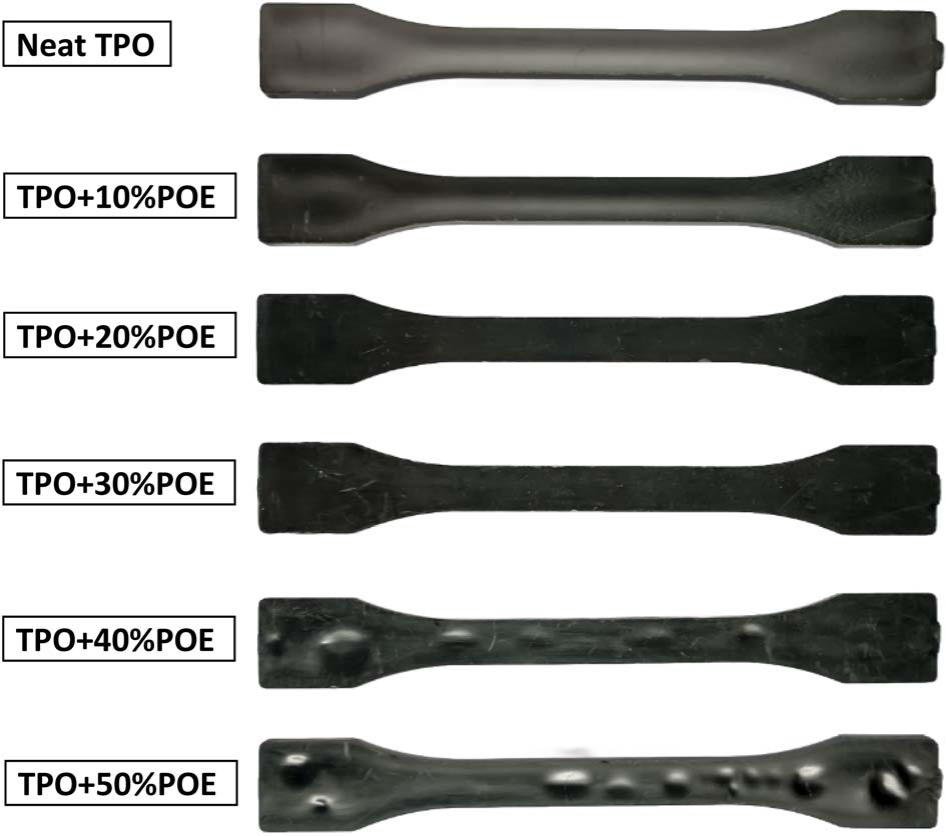
Injection-moulded tensile specimens of TPO and TPO–POE blends, 4 mm thickness, showing sink mark defects on the surface of TPO + 40%POE and TPO + 50%POE specimens.

Fig. 5 shows the tensile modulus and elongation for TPO, POE and TPO–POE blends. The results show that the tensile modulus decreases by 23% to 83% compared with the neat TPO as the concentration of the elastomeric POE increases from 10 wt% to 50 wt%. Specifically, the modulus rapidly decreases from 1850 MPa to about 1456 MPa and 950 MPa with 10 wt% and 20 wt% of TPO substituted by POE respectively, then steadily drops to 636 MPa and 321 MPa for the blends as POE content increases to 30 wt% and 40 wt%, respectively. It is interesting to note that the blends from 10 wt% to 40 wt% have an extraordinary reduction in tensile modulus, which is caused by the reduction of elasticity and stiffness of material blends. Increasing the POE content to 50 wt% decreases the modulus slightly to 250 MPa, which is a small change compared to other formulations. This suggests that the higher increment of POE content in the matrix will reduce the functionality of IP and can easily cause the material to have mechanical failures from such as vibration and thermal changes in automotive applications. In addition, based on the moulding of 4 mm (thickness) tensile test piece for 40 wt% and 50 wt% formulations, a large number of voids inside the material and sink marks on the surface appeared, as shown in Fig. 6. These defects are another indication the addition of POE content above than 40 wt% is not suitable for the moulding material and a large product such as an IP which requires a good appearance. After the addition of POE content more than 50%, the modulus curve shows an estimation of POE content from 60 wt% up to 90 wt% in the matrix, where it gives a small change in modulus due to the lowest material elasticity of POE in the TPO. While, in the actual tensile test, the modulus value for POE is 5.6 MPa.

The strain to failure (Fig. 5) shows an exponential increase with the increasing POE concentration in the matrix. At 10 wt% of POE addition, there is a slight increment from approximately 41% to 80%. Then the strain to failure steadily increases with the addition of POE from 20 wt% to 30 wt%, where the ductility of the blends is 243% and 406% respectively. The curve tends to above 500% strain to failure with the POE addition of 50 wt%, it is expected to reach more than 1000% with a continuous increment of POE content above 65 wt%, while the pure POE shows a strain to failure of more than 2000%. Based on these findings, the addition of POE into TPO will assuredly increase the strain to failure as reported by Zhao *et al.*^14^ and this ductility will make the material difficult to fracture. However, the POE content will reduce the strength of the material and it is significant to define the best ratio of TPO–POE blends. Deciding on the best ratio of TPO–POE blends needs further investigation such as fracture test and DMTA.

**Fig. 7:**
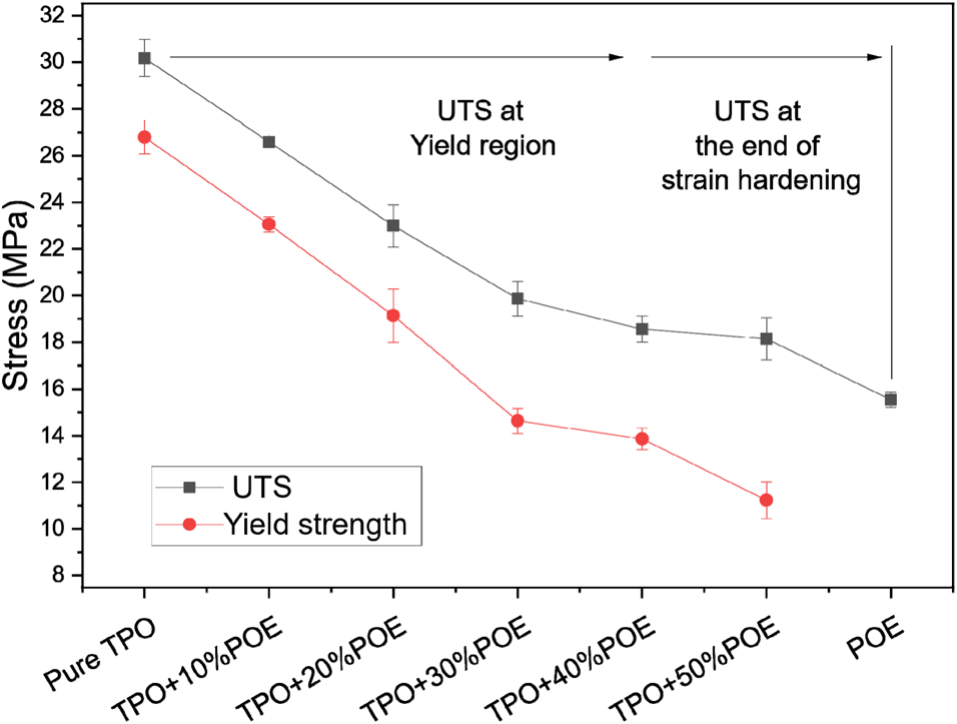
Ultimate tensile strength and yield stress for TPO, POE and TPO–POE blends.

Fig. 7 shows the ultimate tensile strength (UTS) and yield stress for TPO, POE and TPO–POE blends. The ultimate tensile strength is substantially reduced from 30.2 MPa to 19.9 MPa (strength reduction of 34.1%) with the increase of POE in the matrix from 0 wt% to 30 wt%. Increasing the POE content to 50 wt% gives a further slight decrease to 18.2 MPa (a reduction of 46.6%). This value approaches the UTS for POE of 15.6 MPa. In the study of TPO–POE blends ranging from 0 wt% to 30 wt% POE content, a noticeable necking phenomenon occurs as discussed earlier, and the Ultimate Tensile Strength (UTS) values appear within the yield region where the blends undergo permanent deformation. However, with the addition of 40 wt% and 50%, the UTS values are observed at the end of strain hardening due to the synergistic effect between the amount of POE content in the matrix, as explained in Fig. 4. It is suggested that further research is needed to fully understand the mechanisms behind the observed synergistic effect between TPO and POE.

For yield stress, a similar trend is recorded, where the yield stress shows a considerable drop from 26.8 MPa at 0 wt% to 14.6 MPa at 30 wt% (a reduction of approximately 45%), then a slow fall to 11.2 MPa for 50 wt% (a reduction of approximately 23%). The yield stress is important as it indicates the initiation of plastic deformation, so necking will occur, and this is an indication of material failure. For the automotive material, the effect such as static loading, material vibration and temperature variation will reduce the yield stress and then decrease the safety factor of a structure especially at the joint/welding area. Since the reduction of yield stress is considered too high upon the addition of POE into TPO matrix, a careful selection needs to consider when deciding on the amount of elastomer to use to increase impact properties.

### 4.3. Dynamic mechanical thermal analysis (DMTA)

**Fig. 8:**
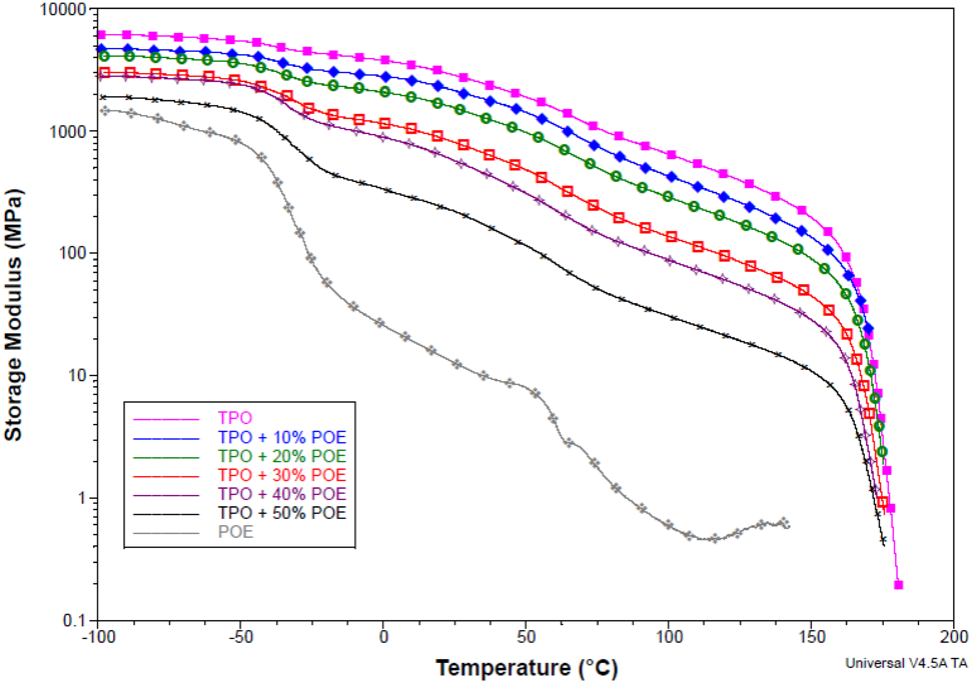
Storage modulus *versus* temperature for TPO, POE and TPO–POE blends.

DMTA was used to characterize the storage modulus, tan delta, and glass transition temperature for TPO, POE and TPO–POE blends as a function of temperature. Fig. 8 is representative of the storage modulus change over the temperature for the materials. The storage modulus decreases with increasing POE content especially at the glass transition temperature of POE and TPO as shown in Table 1. The storage modulus *versus* temperature curves show an intermediate change for 30 wt% and 40 wt% of POE content in the region between −50 °C and 0 °C, while the curve shows a more remarkable change for 50 wt% of POE content. This explains the effect of POE in the matrix, where increasing the POE content will influence the glass–rubber transition of the matrix and the modulus reduction.

**Table 1:** Measured glass transition temperature (*T*_g_), tan *δ* peak and storage modulus values at various temperatures for TPO, POE and TPO–POE blends

TPO/POE	*T*_g_ (°C)			Tan *δ* peak value			Storage modulus (MPa)		
	PP	EPDM	POE	PP	POE	EPDM	at *T*_g_ POE	at *T*_g_ TPO	at 20 °C
POE	—	—	−31.5	—	0.460	—	173	—	15
TPO	25.5	−36.3	—	0.048	—	0.054	4816	2826	3065
TPO + 10%POE	25.3	−35.0		0.059	0.070		3592	2140	2313
TPO + 20%POE	26.8	−33.9		0.060	0.083		2831	1571	1683
TPO + 30%POE	28.1	−31.6		0.070	0.115		1776	762	885
TPO + 40%POE	27.7	−32.7		0.075	0.132		1580	541	642
TPO + 50%POE	—a	−30.8		—a	0.180		737	—a	242

a The peak point does not appear.

**Fig. 9:**
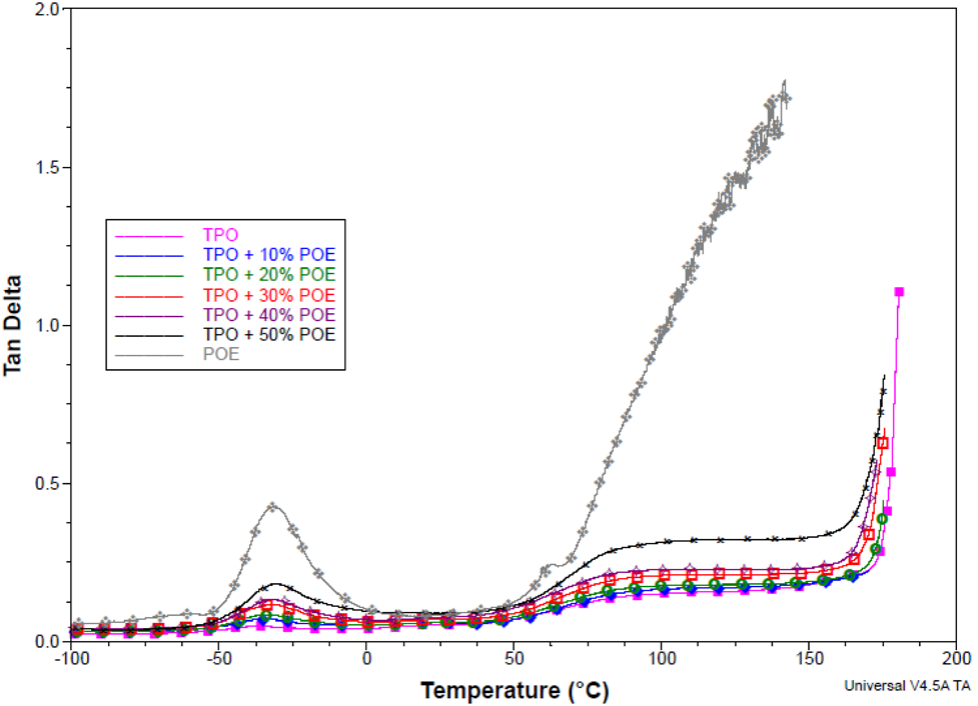
Damping tan delta *versus* temperature for TPO, POE and TPO–POE blends.

It is important to analyse the glass transition temperature of materials, especially to characterise the material to work in any temperature change. Fig. 9 presents the damping tan delta against temperature for the TPO, POE and TPO–POE blends. Two glass transition temperatures were identified for pure TPO, at 25.5 °C and −36.3 °C as shown in Table 1. The TPO is a high flow 25% talc filled EPDM rubber modified polypropylene compound. The glass transition value shows that TPO has been developed for automotive interior material with good impact resistance and can work under extreme low temperatures, *e.g.* below −30 °C. Based on the temperature data for pure TPO, the glass transition temperature (*T*_g_) for EPDM rubber is −36.3 °C, while the value for PP is 25.5 °C, see Fig. 9. Thus, in the addition of POE content into TPO matrix, the glass transition temperature is an important material property to consider, where it must be above −30 °C. In Table 1, the addition of POE content decreases the glass transition temperature, and POE shares the glass transition point with EPDM rubber as expected. Fig. 9 shows that the addition of 10 wt% and 20 wt% of POE content reduced slightly the glass transition temperature, to −35.0 °C and −34.0 °C, respectively. Meanwhile the addition of POE content above 30 wt%, the glass transition temperature is recorded below −33 °C and near to the accepted working condition. Therefore, the addition of 10 wt% and 20 wt% of POE content would be recommended for the IP to work with airbag application.

Fig. 9 also indicates the damping performance through the value of the tan delta peak along the temperature transition. The tan delta values for PP and POE are 0.048 and 0.46 respectively, while the value for EPDM is similar to the trend seen in TPO, where the value is unaffected by the stiffness of TPO. The presence of talc in TPO reduces the tan delta peak value as the relatively high loading of filler (25%) restricts the mobility of the polymer chains and hence reduces the damping.^14^ The addition of POE increased the mobility and hence the tan delta peak value increased accordingly. Based on the curve, the damping peak show a major change upon the addition of POE content above 30 wt%, while the POE content with 10 wt% and 20 wt% shows a minor change below tan delta of 0.1.

### 4.4. Charpy test

**Fig. 10:**
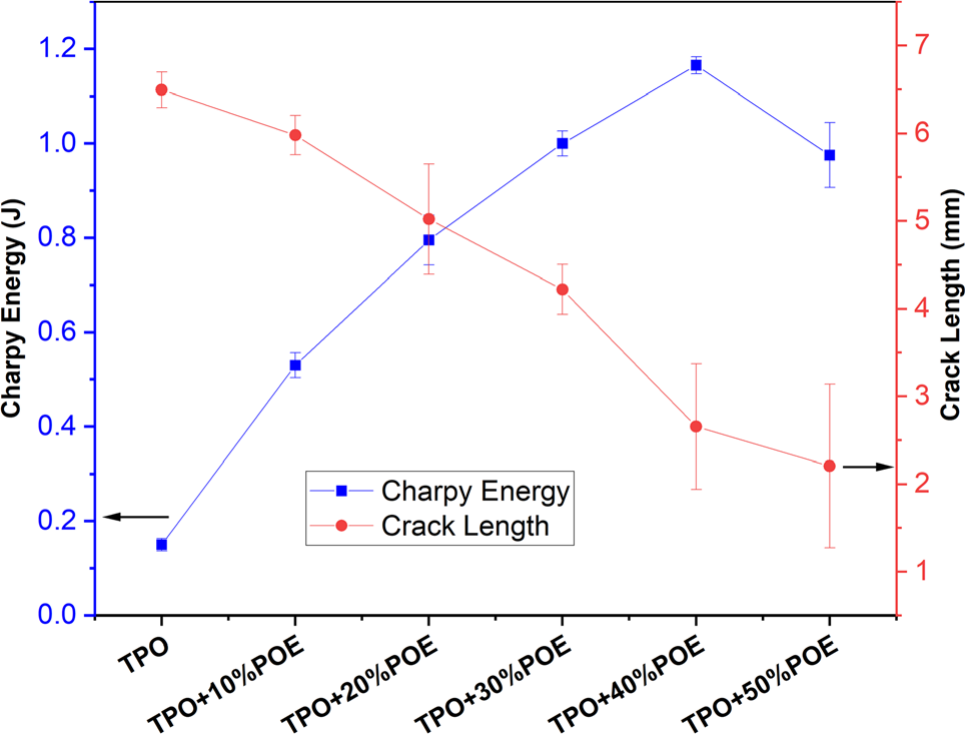
Charpy impact energy for TPO and TPO–POE blends.

Charpy tests were conducted to evaluate the amount of energy (as shown in Fig. 10) absorbed during impact. The energy for pure TPO is 0.14 J and the test piece broke entirely to create a flat fracture surface. The absorbed energy increases with the addition of POE content and reduced the distance of fracture length. The addition of 10 wt% of POE content led to a rapid increase in the energy absorption to 0.54 J, while the addition of 20 wt% to 40 wt% POE content cause a further increment of the energy absorption from 0.54 J to 1.145 J. The maximum energy is recorded at 40 wt% of POE content, and the energy is reduced to 0.9 J with the addition of 50 wt% of POE content. The test pieces produced a partial break rather than fracture entirely, due to TPO–POE blends exhibiting more deformation to absorb energy as the load is applied, and the final crack length reduced with increasing POE content. The crack length values of the addition of pure TPO, 10 wt%, 20 wt%, 30 wt%, 40 wt% and 50 wt% for the absorbed energy at break are 6.73 mm, 6.23 mm, 5.75 mm, 4.55 mm, 2.13 mm and 1.25 mm respectively. Thus the addition of POE content in TPO matrix (as an automotive material) will increase the energy absorption and increase the material resistance to fracture under an impact.

### 4.5. Hardness test

**Fig. 11:**
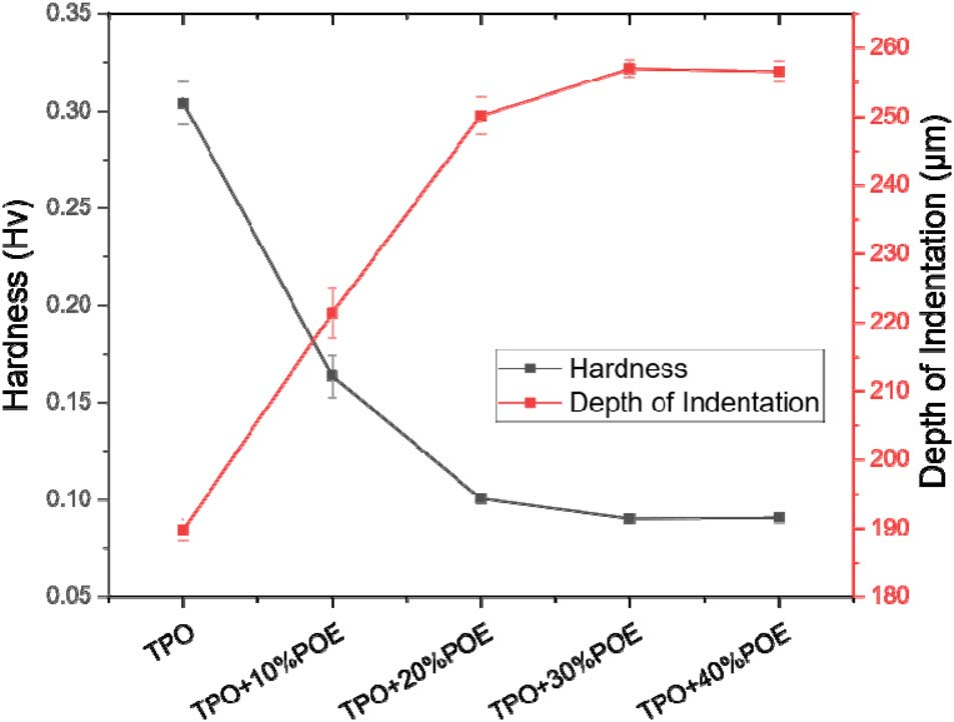
Vickers hardness test results for TPO and TPO–POE blends. Note that TPO + 50%POE was too soft to be measured.

Fig. 11 shows the Vickers hardness numbers (VHNs) for TPO and TPO–POE blends. These are soft materials because of the elastomer content. For neat TPO, the hardness is 0.3 Hv at an indentation depth of 190 μm and the hardness value decreases with increasing POE content. With the addition of 10 wt% and 20 wt% of POE content, the hardness value drops about 46% and 67% respectively, while the indentation of depth for both blends is found to be at 221 μm and 250 μm. With 20 wt% of POE content and above, the indentation of the depth and hardness of the materials show a small change and is expected to reach a plateau. When an indenter is pressed into the TPO–POE blend, the material will exhibit both elastic and viscous responses. With the increment of POE in the matrix, the material becomes soft and increases the *d*^2^ value, as well as the elastic response, causing it to bounce back rapidly when the indenter is removed. The 50 wt% POE material is too soft for the Vickers hardness machine to measure. These results indicate that POE can be successfully used to achieve the desired reduction in hardness of TPO and is important to achieve suitable flexibility of the airbag housing material for it to work well during airbag deployment.

### 4.6. Fracture test

The fracture resistance and crack growth for TPO and TPO–POE blends were measured.^28,29^ Fracture and compliance tests were conducted to develop a J-crack growth resistance graph using the multi-specimen J-integral approach.^30–34^

#### 4.6.1. Pre-tests

**Fig. 12:**
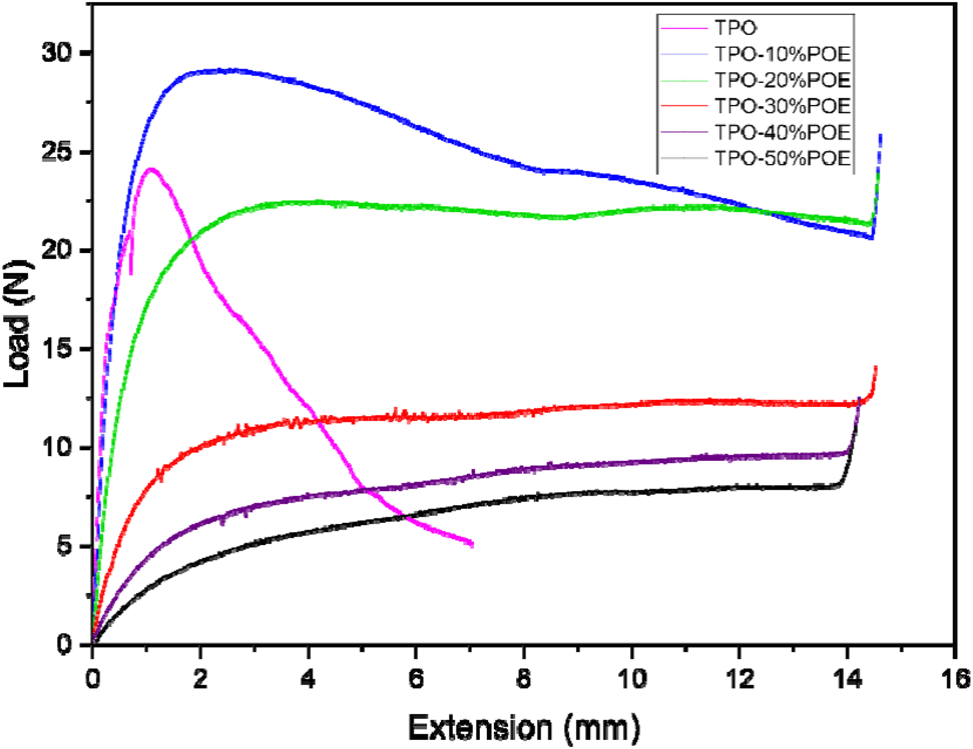
Load *versus* extension curve of TPO and TPO–POE blends during SENB test.

**Fig. 13:**
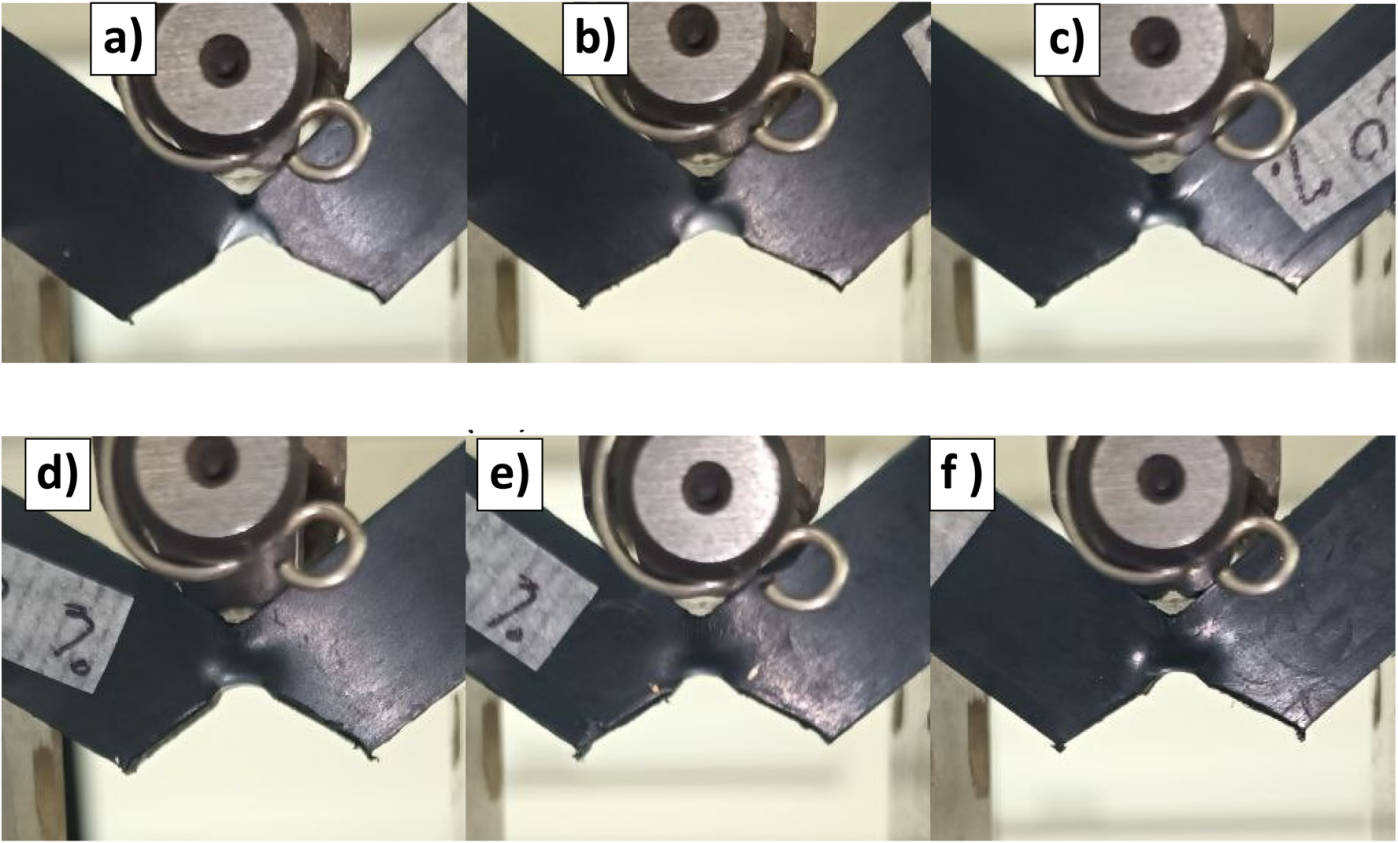
Photographs of SENB samples after the pre-tests for pure TPO (a), TPO + 10%POE (b), TPO + 20%POE (c), TPO + 30%POE (d), TPO + 40%POE (e), and TPO + 50%POE (f).

**Fig. 14:**
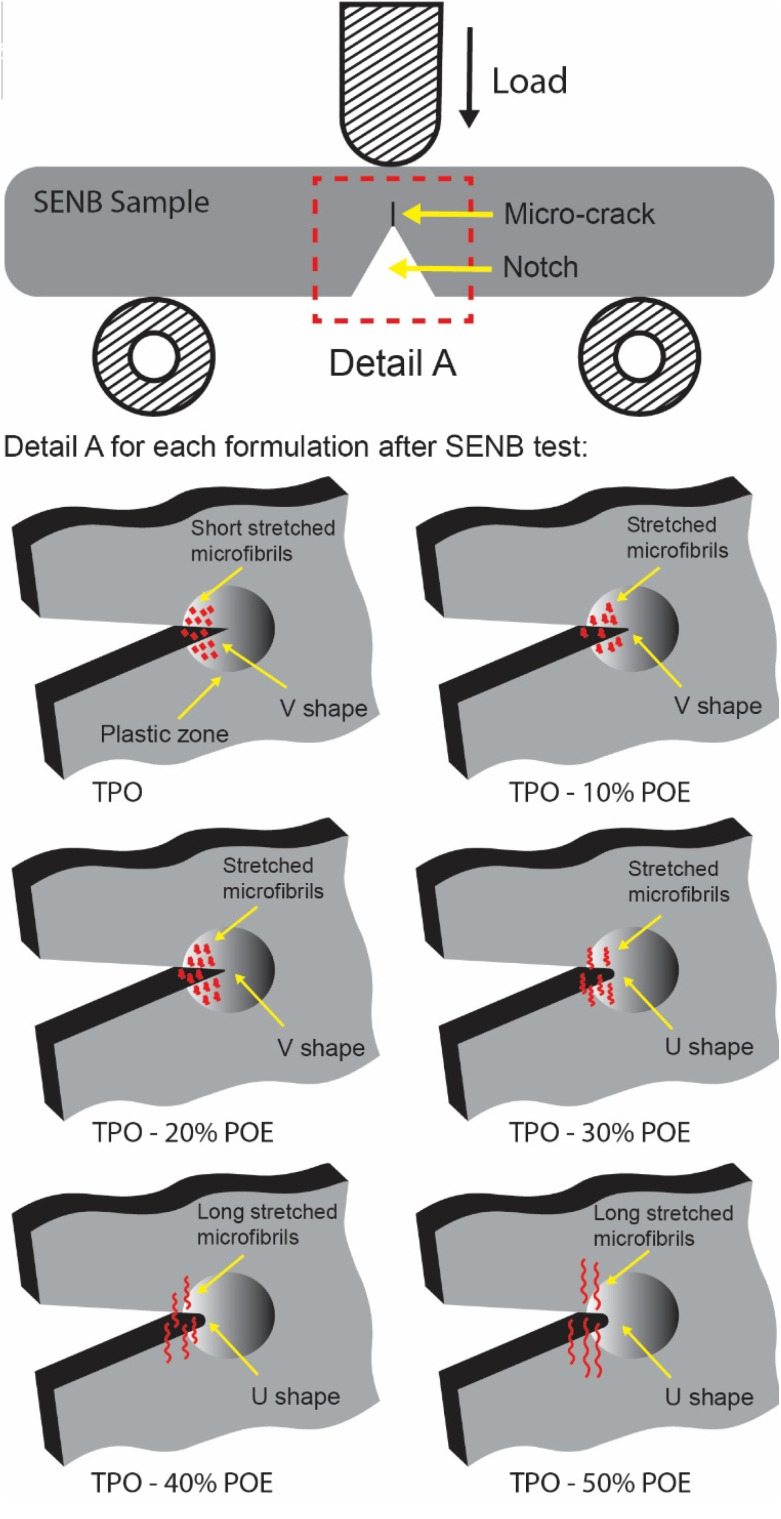
The schematic illustrates how the addition of POE amount to TPO changes the crack tip region from V shape to U shape after SENB tests.

Fracture testing polypropylene-type materials is complex and challenging due to the difficulties encountered in the experimental measurement of the crack resistance.^28,31,33,35^ Therefore, a dummy test was carried out for reach material to determine the maximum load, and hence enable the load required to generate the correct amount of crack growth in the SENB tests to be estimated. The load *versus* extension curves are shown in Fig. 12, and indicate that the modified polymers exhibit a large viscoelastic displacement compared with pure TPO when enduring a non-linear deformation (time-dependent strain). The increment of POE in the matrix allows the absorption of energy and compensates for the crack to propagate with a higher extension. However, due to the flexibility of the modified TPO, the maximum load is reduced with the increment of POE. The dummy test was stopped when the specimen touched the roller support as shown in Fig. 13. The images in Fig. 13 show that the geometry of the crack tip changed from a V-shape to a U-shape with the increment of POE content as well as the reduction in crack length and white mark content. This result indicates that the increment of POE content causes crack tip blunting where the notch tip is highly deformed by plasticity of the polymer.^31^ In Fig. 14, a schematic demonstrates the effect of introducing POE to TPO on the formation of the crack tip region. This alteration shifts the crack tip shape from a V shape to a U shape in comparison to pure TPO, a change observed subsequent to the SENB test. This transition significantly advances the reinforcement and yielding mechanism, attributing it to the elongation of microfibrils.

#### 4.6.2. Pre-test fractography

**Fig. 15:**
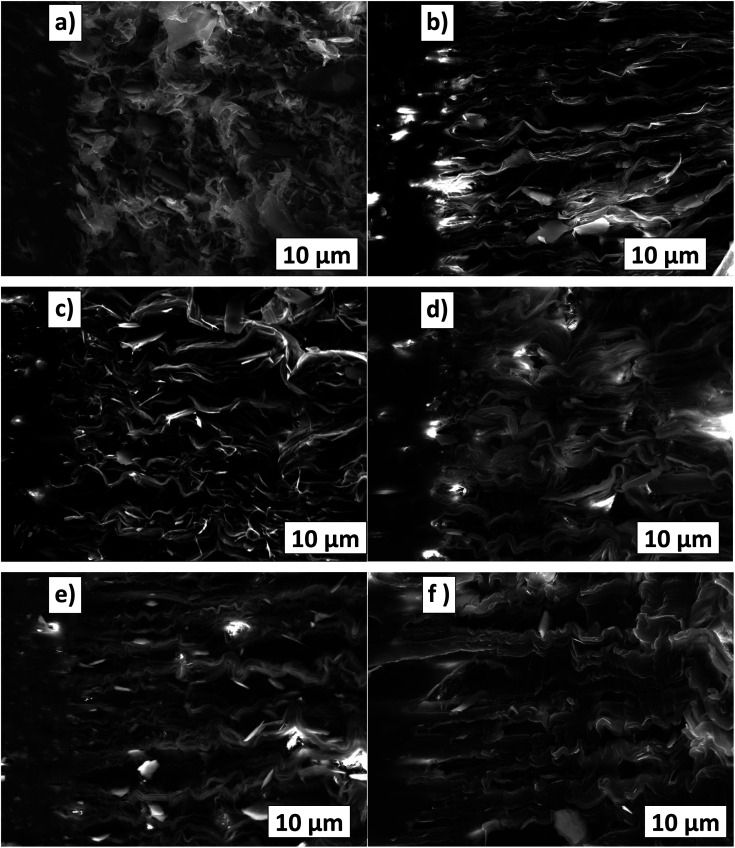
Fracture surface from SENB tests (after pre-tests) for pure TPO (a), TPO + 10%POE (b), TPO + 20%POE (c), TPO + 30%POE (d), TPO + 40%POE (e), and TPO + 50%POE (f).

To understand the deformation and fracture of the TPO–POE blends, a fractographic analysis was carried out at the final crack region after the SENB pre-tests. The large strains applied show clearly the deformation of the polymers and enable greater understanding of the SENB results presented later (where the surfaces may be affected by the dye used to highlight the crack growth). After the pre-test, the TPO (see Fig. 15(a)) has short stretched microfibrils and some talc at the final crack region. At the end of the notch region, the non-uniform distribution of stretched microfibrils is observed in the crack direction. Moreover, the presence of talc act as an internal defect in the TPO and promote debonding contribution to plastic deformation.^36^ Hence, these mechanisms reduced the fracture resistance of the TPO.

**Fig. 16:**
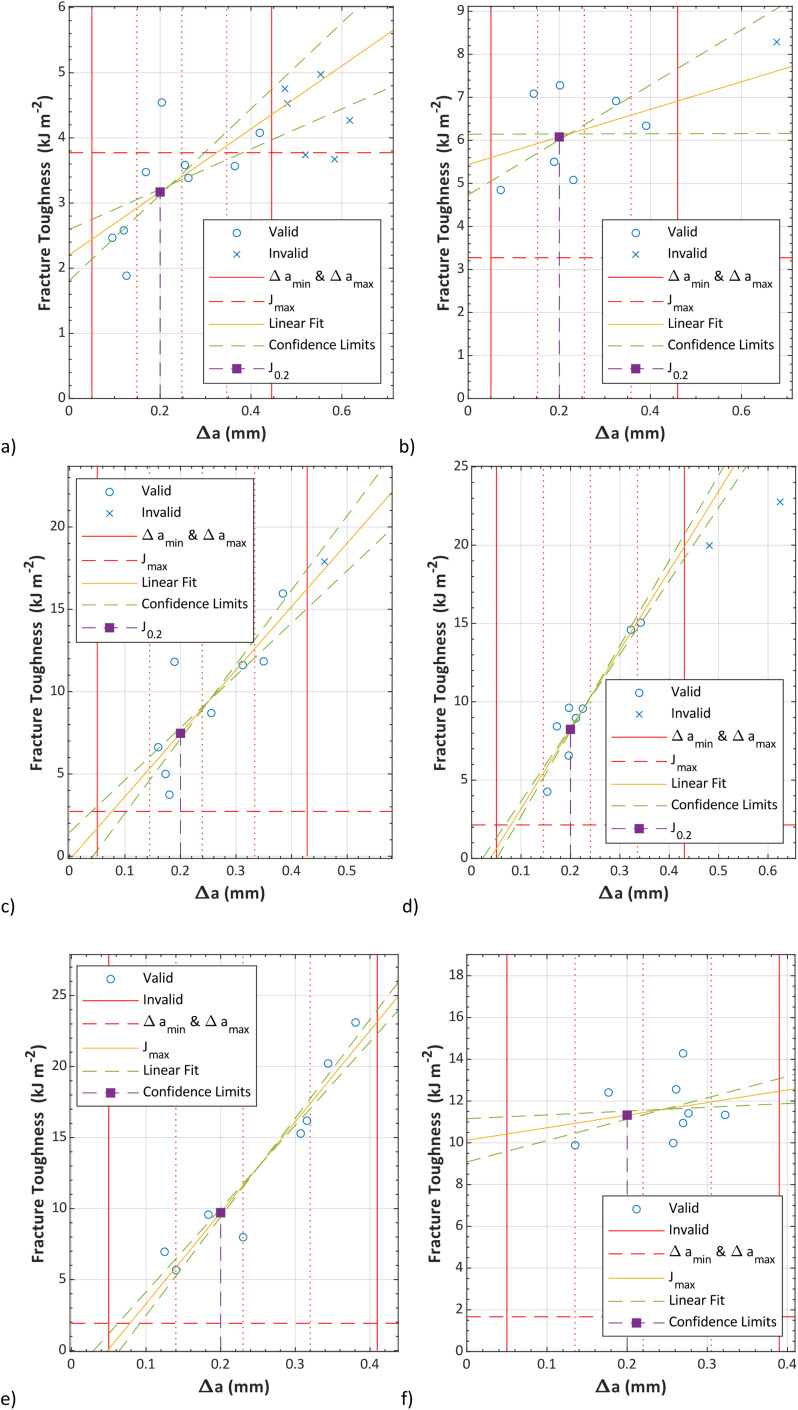
Fracture resistance for pure TPO (a), TPO + 10%POE (b), TPO + 20%POE (c), TPO + 30%POE (d), TPO + 40%POE (e), and TPO + 50%POE (f).

With the addition of POE in the TPO, the surface textures of fracture surfaces as shown in Fig. 15(b)–(f) have altered. Under quasi-loading, the POE microfibrils tend to elongate further and some microfibrils can be seen as the white colour (crazing) at crack initiation. With the increasing ratio of POE in the matrix, the amount of stretched microfibrils which elongate appears to increase. For 10 wt% and 20 wt% of POE ratio, the microfibrils become more oriented and elongated in the crack growth direction. This is attributed to a strong contribution of a long molecular chain of POE at the beginning of crack propagation. Thus, this mechanism leads to an increase in fracture resistance as shown in Fig. 16. Furthermore, with the addition of 30 wt% to 50 wt% POE content into the matrix, the evolution of the stretched microfibrils becomes longest due to highly yielding behaviour in plastic deformation of POE. However, TPO with 30 wt% of POE content shows some lacking orientations due to the transition from a sharp crack tip to a blunting mechanism as demonstrated in Fig. 13. An apparent blunting morphology of the crack tip is visibly formed with the increment of POE content to 40 wt% and 50 wt% due to the alignment of stretched microfibrils. For the 50 wt% of POE content, the microfibrils clearly elongated across the entire fracture surfaces.

**Fig. 17:**
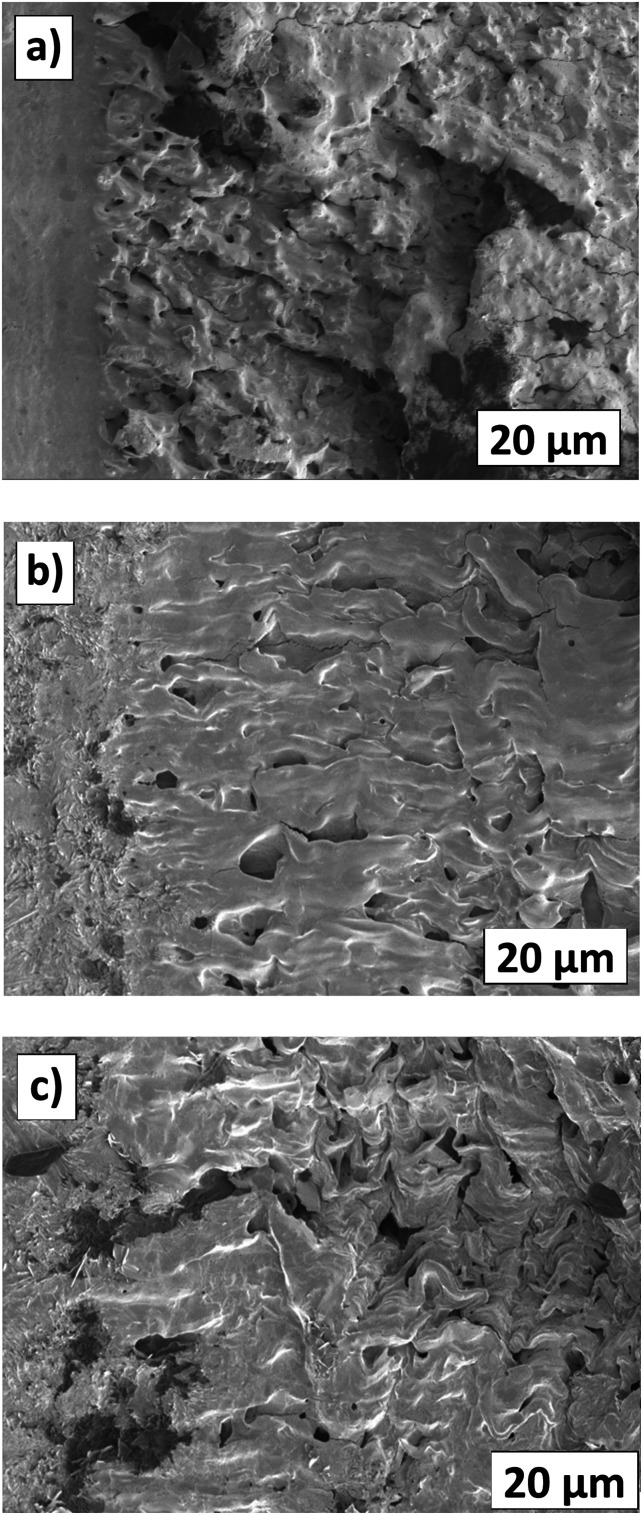
Fracture surface from SENB tests (after pre-tests) for pure TPO (a), TPO + 20%POE (b), and TPO + 40%POE (c).

The fracture surface in Fig. 17(a) shows that pure TPO contains a large number of randomly-distributed micro-voids and un-oriented fracture lines between micro-voids in the plastic zone area. Thus, these cavitations formed from nanometer to micrometer sizes^37^ are expected to speed up material deformation. In this sense, it can be said that the micro-voids inside the material increase the concentrated stress and produce micro fracture lines to connect with other micro-voids in the neat TPO, which were formed during the crack propagation process.^38^ It seems that the crack advances when the fibrils at the trailing edge of the craze cease to extend and rupture.^39^ This behaviour leads to fracture energy reduction, the increment of stress whitening area and produces unstable crack patterns. Fig. 17(b) and (c) illustrate the fracture surface of 20 wt% and 40 wt% TPO–POE blends, it shows that the addition of POE content provides better processability thus compensating the number of micro-voids and microfracture line inside the matrix. Under a quasi-static loading, the addition of POE content increased the fracture resistance due to the strong yielding of the POE microfibrils between micro-voids, thus increasing the crack propagation energy and decreasing resistance to crack initiation.^19^ The yielding phenomenon can be seen in Fig. 4, where the addition of more POE increased the plastic deformation region as POE in the matrix behaves like a rubber network. This finding suggests that a pure TPO is unsuitable for use in an airbag system and this material requires modification with POE to absorb the energy during the airbag deployment, and to resist material failure in undesirable locations or over-fracture.

**Fig. 18:**
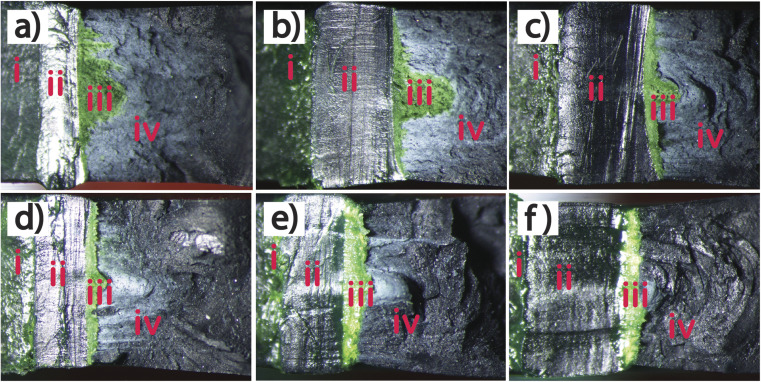
Fracture surface from SENB tests (after cryo-fracturing) for pure TPO (a), TPO + 10%POE (b), TPO + 20%POE (c), TPO + 30%POE (d), TPO + 40%POE (e), and TPO + 50%POE (f). The image can be divided into regions of the machined notch area (i), pre-crack region (ii), crack growth (dyed green) (iii), and final brittle (cryo) fracture (iv).

Fig. 18 shows topographies of fracture surfaces for pure and modified TPO. The topology of the fracture surfaces of each test piece is divided into machined notch area, pre-crack region, crack growth and final brittle fracture. In the pre-crack region, the length of pre-crack (*a*_0_) for TPO is smaller compared to TPO with POE content due to pure TPO material being hard to cut using the sliding method, then it is difficult to control the pre-crack length. Meanwhile, with the increment of POE content, the SENB test piece is easier to cut and control the pre-crack due to the increased flexibility of the material, however, the sliding process needs to control to avoid over or slanting cut. The final crack length (*a*_f_) is occupied by a green area due to an uneven and rough surface formed during the fracture test. This area indicates the deformation of stable crack growth which promote low yield stress and high toughness.^22^ For pure TPO, a half-moon feature was formed on the fracture surface. With the POE content of 10 wt% and 20 wt%, the half-moon becomes smaller as shown in Fig. 18(b) and (c). A straight crack growth appeared for the sample with the POE content above 40 wt% in the matrix. It suggests that the samples with POE contents of 35 wt% and above in the matrix are deformed to produce a blunting crack tip due to the shift of the brittle–ductile transition point.^21^ This behaviour can be seen in Fig. 18(e) and (f), where the POE content of 40 wt% and 50 wt% in the matrix to show the sign of ductility all along the thickness of the sample.

#### 4.6.3. J-integral

As mentioned earlier, each formulation required at least 7 samples to be tested, however, the actual SENB test for each formulation required more than eight samples due to the sample sliding problem (these results are not included in the *δJ*/*δ*Δ*a* curve) and the crack exceeding than maximum crack growth (Δ*a*_max_). Since many samples for each formulation required single-point data, cryo-fracturing and deforming the ductile material at a slow speed, this testing consumed a lot of time and was challenging to obtain a valid result. Based on Fig. 16, pure TPO shows six samples beyond the maximum crack growth (Δ*a*_max_) due to unstable crack conditions. The unstable crack growth can be explained by the existing micro-voids on the fracture surface as shown in Fig. 17(a). The existing micro-voids inside the matrix promote unstable crack propagation. Due to this reason, this TPO is considered unsuitable for use as the instrument panel or the airbag housing (also commonly made of TPO).

To quantify the fracture energy, the fracture toughness (*δJ*) *versus* Δ*a* curves according to the multi-specimen J-integral procedure were constructed through the power-law to the experimental values, with 90% confidence intervals as illustrated in Fig. 16. It is worth noting that the POE content improved the toughness of TPO, particularly to increase fracture energy. A point on a power law curve corresponding to *J*_0.2_ for each graph is indicated. Based on the *J*_0.2_ results, pure TPO shows at the lowest fracture resistance as the energy is recorded at 3.17 kJ m^−2^. With 10 wt% of POE, the *J*_0.2_ value almost doubles to 6.08 kJ m^−2^. This finding supports the statement indicating that the POE provides a dual function by reinforcing and toughening,^15^ due to the long chain branching structure of POE improves its toughness. Furthermore, the addition of POE content fills the micro-voids in the matrix and provides strong shear yielding. The fracture resistance for the addition of POE content for 20 wt%, 30 wt%, 40 wt% and 50 wt% shows a gradual increase, with *δJ*/*δ*Δ*a* values at Δ*a* = 0.2 mm of 7.42 kJ m^−2^, 8.24 kJ m^−2^, 9.71 kJ m^−2^ and 11.32 kJ m^−2^ respectively. The higher energy leads to higher fracture toughness and higher resistance to crack growth, which can be equated to higher material performance.^30^ However, for the addition of 40 wt% and 50 wt% POE content, sink mark defects appeared during the moulding process due to different cooling times between TPO and POE as illustrated in Fig. 6. It suggests that the modification of POE content into the matrix can be added in between 10 wt% to 30 wt% without a significant decrease in the quality of the moulded component.

The ESIS procedure^28^ suggests that *J*_0.2_ is valid if (1) at least one *J* − Δ*a* point falls between the minimum and maximum of crack growth and (2) *J*_0.2_ is below *J*_max_. Based on the results, only TPO meets these requirements, as the *J*_0.2_ values for TPO containing POE are above *J*_max_. as shown in Table 2. The *J*_max_ value for each formulation is reduced due to the reduction of yield stress with the increment of POE in the matrix, as shown in Table 2. However, the latest ASTM standard^29^ does not require *J*_0.2_ to be below *J*_max_, and many articles in the literature exclude the *J*_max_ value in the fracture toughness (*δJ*) *versus* Δ*a* curves.

**Table 2:** Yield stress and fracture resistance values for TPO and TPO–POE blends

Material	TPO	TPO + POE (10%)	TPO + POE (20%)	TPO + POE (30%)	TPO + POE (40%)	TPO + POE (50%)
Yield stress, (MPa)	24.37	20.93	17.33	13.47	12.02	10.33
*J*_max_, (kJ m^−2^)	3.77	3.27	2.73	2.14	1.92	1.67
*J*_0.2_, (kJ m^−2^)	3.17	6.08	7.42	8.24	9.71	11.33
Confidence interval (max), (kJ m^−2^)	3.21	6.15	7.81	8.34	10.00	11.52
Confidence interval (min), (kJ m^−2^)	3.13	6.01	7.13	8.14	9.43	11.13

According to Hale,^31^ a *J*–*R* curve approach is not valid for highly ductile materials such as the modified TPO with higher POE content as these are extremely tough and yielding occurs at the crack tip rather than crack propagation. Meanwhile, Williams^31^ mentions that characterising very tough material requires a very thick specimen, but such a specimen would not be representative of the thin material that fractures in the instrument panel application.

A correction can be applied to account for the blunting crack tip (semi-circular) formed due to the material's ductility.^31^ Thus the new crack growth meets the following equations:7
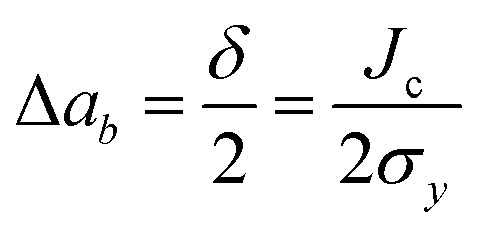

8 *J*_c_ = 2*σ*_*y*_Δ*a*_*b*_

Where *σ*_*y*_ is the yield strength, *δ* is the crack opening displacement, *a*_*b*_ is the new crack growth. This correction seems not to work well with the TPO modified with more than 30 wt% of POE, as the fracture resistance reduces to half and does not lie within the multiple J-integral points on the graph. This suggests that the ESIS procedure^28^ is mainly developed for less tough materials and requires some further correction to determine the fracture energy of very tough or highly-ductile materials with crack tip blunting. Nevertheless, the fracture energy obtained is considered valid as the guidelines in ESIS TC4-J^28^ and ASTM D6068-10 ^29^ are applied and the results are realistic according to current findings, *i.e.* SEM analysis, impact test and literature review.

The overall result shows that the ESIS TC4-J^28^ and ASTM D6068-10 ^29^ procedures are effective ways to calculate the fracture energy for the IP-airbag material. This testing is considered as best practice to understand the behaviour of pure and modified TPO. Some limitations of the TPO material were successfully identified, indicating that POE modification is required to resist such an unwanted failure and over-fracture for the airbag application. Nevertheless, more experimental evidence is required such as *J*_max_ correction, blunting crack tip issues and to apply this in real instrument panel tests in order to increase the safety of the airbag deployment in a vehicle.

## 5. Conclusion

The performance of vehicle instrument panel (IP) material is evaluated to understand the fracture toughness and mechanical properties through fundamental testing to ensure the material resists failure in undesired locations or over-fracture during airbag deployment. A typical thermoplastic olefin (TPO) was used, and modified with polyolefin elastomer (POE) to enhance its flexibility and impact properties. A multi-specimen procedure was used to determine the fracture resistance of the materials, and the following conclusions can be determined:

(i) Neat TPO is too brittle for the IP-airbag application. The addition of POE increases the fracture resistance and the modified material becomes suitable for the application.

(ii) The addition of POE content above 30 wt% caused crack blunting crack tip, which would require further corrections to apply the J-procedure.

(iii) The addition of POE promotes crazing, and the stretched microfibrils at the crack tip become more extended with increasing POE content due to the greater extensibility of the material. This increases the fracture resistance.

(iv) The fracture resistance of neat TPO was relatively low due to the presence of micro-voids and the formation of short microfibrils. However, the incorporation of POE content improved fracture resistance by strengthening the yielding of POE microfibrils surrounding any micro-voids. This resulted in an increase in the energy required for crack formation and an increase in the resistance to crack initiation.

(v) With 10 wt% of POE content in TPO, the *J*_0.2_ value almost doubles, while the addition of 50 wt% POE content, the *J*_0.2_ value becomes almost quadruple.

Fundamental mechanical tests were also carried out, and the following conclusions can be determined:

(i) The tensile modulus, ultimate tensile strength and yield stress decreased slightly with the increase of POE content, while the ductility increased dramatically.

(ii) The addition of POE increased the Charpy energy absorption during an impact.

(iii) The storage modulus and glass transition temperature (*T*_g_) values decreased slightly with increasing POE content, while the damping increased.

Based on these tests, it can be concluded that the addition of POE content between 10 wt% and 20 wt% enhances the ductility and toughness properties of the TPO–POE blends to improve fracture resistance without detrimental effects on other properties. Thus, this modification is recommended for instrumental panel applications.

These results show how elastomers can improve the properties of interior automotive polymers, especially when subjected to airbag deployment. Further work is required to evaluate dynamic fracture performance with operating temperature. This is a critical study to determine material properties for interior car components and thus improve the robustness of the airbag structure during the deployment process to ensure passenger safety.

## Conflicts of interest

There are no conflicts to declare.

## Supplementary Material
